# IGF2 Promotes Growth of Adrenocortical Carcinoma Cells, but Its Overexpression Does Not Modify Phenotypic and Molecular Features of Adrenocortical Carcinoma

**DOI:** 10.1371/journal.pone.0103744

**Published:** 2014-08-04

**Authors:** Marine Guillaud-Bataille, Bruno Ragazzon, Aurélien de Reyniès, Claire Chevalier, Isabelle Francillard, Olivia Barreau, Virginie Steunou, Johann Guillemot, Frédérique Tissier, Marthe Rizk-Rabin, Fernande René-Corail, Abir Al Ghuzlan, Guillaume Assié, Xavier Bertagna, Eric Baudin, Yves Le Bouc, Jérôme Bertherat, Eric Clauser

**Affiliations:** 1 Paris Cardiovascular Center, Institut National de la Santé et de la Recherche Médicale U970, Université Paris Descartes, Paris, France; 2 Département de Biologie Hormonale, Assistance Publique Hôpitaux de Paris, Hôpital Cochin, Paris, France; 3 Institut Cochin, Institut National de la Santé et de la Recherche Médicale U1016, Centre National de la Recherche Scientifique UMR8104, Université Paris Descartes, Paris, France; 4 Programme Cartes d'Identité des Tumeurs, Ligue Nationale Contre le Cancer, Paris, France; 5 Département d'Endocrinologie, Assistance Publique Hôpitaux de Paris, Hôpital Cochin, Paris, France; 6 Institut National de la Santé et de la Recherche Médicale U938, Université Pierre et Marie Curie, Paris, France; 7 Laboratoire d'explorations fonctionnelles endocriniennes, Assistance Publique Hôpitaux de Paris, Hôpital Armand Trousseau, Paris, France; 8 Service d'Anatomie Pathologique, Assistance Publique Hôpitaux de Paris, Hôpital Pitié-Salpétrière, Université Pierre et Marie Curie, Paris, France; 9 Département de Biologie et Pathologie Médicales, Institut Gustave Roussy, Villejuif, France; 10 Département d'Imagerie Médicale, Médecine nucléaire, Institut Gustave Roussy, Villejuif, France; Columbia University, United States of America

## Abstract

*Insulin-like growth factor 2 (IGF2)* overexpression is an important molecular marker of adrenocortical carcinoma (ACC), which is a rare but devastating endocrine cancer. It is not clear whether *IGF2* overexpression modifies the biology and growth of this cancer, thus more studies are required before *IGF2* can be considered as a major therapeutic target. We compared the phenotypical, clinical, biological, and molecular characteristics of ACC with or without the overexpression of *IGF2*, to address these issues. We also carried out a similar analysis in an ACC cell line (H295R) in which *IGF2* expression was knocked down with si- or shRNA. We found no significant differences in the clinical, biological and molecular (transcriptomic) traits between IGF2-high and IGF2-low ACC. The absence of *IGF2* overexpression had little influence on the activation of tyrosine kinase pathways both in tumors and in H295 cells that express low levels of IGF2. In IGF2-low tumors, other growth factors (FGF9, PDGFA) are more expressed than in IGF2-high tumors, suggesting that they play a compensatory role in tumor progression. In addition, *IGF2* knock-down in H295R cells substantially impaired growth (>50% inhibition), blocked cells in G1 phase, and promoted apoptosis (>2-fold). Finally, analysis of the 11p15 locus showed a paternal uniparental disomy in both IGF2-high and IGF2-low tumors, but low *IGF2* expression could be explained in most IGF2-low ACC by an additional epigenetic modification at the 11p15 locus. Altogether, these observations confirm the active role of IGF2 in adrenocortical tumor growth, but also suggest that other growth promoting pathways may be involved in a subset of ACC with low IGF2 expression, which creates opportunities for the use of other targeted therapies.

## Introduction

Insulin-like growth factor 2 (IGF2) is a polypeptide hormone with high homology to proinsulin and IGF1. IGF2 is an autocrine, paracrine and endocrine factor which binds with high affinity to membrane-bound tyrosine kinase receptors [1]. Insulin and IGF1 are specific for their cognate receptors, whereas IGF2 has similar affinities for the IGF1 receptor (IGF1R) and the short isoform of the insulin receptor (INSR). The binding of IGF2 to IGF1R and INSR activates the PI3kinase/Akt and MAP kinase pathways and regulates cell division and survival [2]. In addition, IGF2 binds to several specific IGF binding proteins (IGFBP) and the IGF2/mannose6P receptor, which all regulate the bioavailability of IGF2 [3].

IGF2 exerts its proliferative effects in many tissues and its physiological action has been demonstrated primarily during fetal growth [4]. Accumulating data indicate that IGF2 is produced in large amounts by subsets of embryonic tumors, including Wilms tumors, rhabdomyosarcoma [5], hepatoblastoma and neuroblastoma [6]. Subsets of adult cancers also overexpress *IGF2*, including 20% of hepatocarcinoma [7], 40% of colon carcinoma, 90% of liposarcoma [8], and also adrenocortical carcinoma [9].

The production of IGF2 in adult adrenocortical cancer (ACC) and its role in adrenocortical malignant tumorigenesis has been extensively investigated [10]. Unlike adrenocortical adenoma (ACA) which is frequent (2 to 3% of the general population) and is often found incidentally, ACC is extremely rare and has a very poor prognosis [11]. This cancer is revealed either by its tumorigenic progression (compression, metastasis) or more often, by the symptoms of the over-production of steroids (cortisol and androgens, but also estrogens and aldosterone) [12]. Many studies have attempted to identify markers for the diagnosis and prognosis of these tumors. Among these markers, the overexpression of *IGF2* has been recognized for over 20 years [9]. *IGF2* overexpression has been extensively analyzed [13]-[15]. Both IGF2 mRNA (10 to 20 fold higher than normal adrenal gland or ACA) and protein (8 to 80 fold higher than normal adrenal gland or ACA) are overexpressed in 90% of ACC, with a strong correlation between mRNA and protein abundance [13]. *IGF2* overproduction is the consequence of paternal uniparental disomy (pUPD) of the *IGF2* locus at the 11p15.5 region or a loss of imprinting (LOI) of the maternal allele [14], [15]. The ACC cell line H295R recapitulates the IGF2 abnormalities of most ACC, with a high abundance of IGF2 mRNA and protein [16], a low abundance of H19 and CDKN1C suggesting 11p15 pUPD, and *TP53* mutation [17]. This cell line is therefore a good model to study the role of IGF2 in the development of these tumors. The proliferation of this cell line is inhibited by anti-IGF2 [16] and anti-IGF1R antibodies [18], and by specific inhibitors of IGF1R [19]. Several pangenomic transcriptome microarray analyses, including ours, have shown recently that *IGF2* is the most differentially expressed gene between malignant and benign adrenocortical tumors [20]–[23]. Conversely, the overexpression of *IGF2* specifically in the adrenal cortex of transgenic mice has no major tumorigenic effect [24], [25]. However, several questions remain unsolved: when and how is IGF2 important in ACC development? Are ACC that do not overproduce IGF2 phenotypically different from those overproducing this growth factor? Is this growth factor and its signaling pathway a good therapeutic target in ACC?

In the present study, we compared the phenotypic traits and transcriptome of ACC tumors and cells with or without *IGF2* overexpression. Our findings shed new light on the role of IGF2 in ACC tumor progression.

## Material and Methods

### Patients

Samples of sporadic adrenal tumors were obtained from 140 patients mostly followed up at Cochin hospital (Paris, France) between 1993 and 2008 and within the Comete network. The median follow-up period was 51 months. Among these tumors, 87 were diagnosed as ACA (Weiss <3), and 53 as ACC (Weiss ≥ 3) according to criteria described by Weiss *et al.* [26]. A transcriptome analysis was reported previously for 47 adenoma and 29 ACC; 23 overexpress *IGF2* and six do not [21]. *IGF2* expression was measured by quantitative RT-PCR, as previously described [21], identifying two subgroups of ACC with or without overexpression of *IGF2:* ACC IGF2-high, n = 43 (81%), and ACC IGF2-low, n = 10 (19%). The clinical, histopathological, and biological features of these tumors are summarized in Table 1. The Ki67 value was determined by immunohistochemistry. The LOH status of 17p13 was inferred from the study of microsatellite markers [27]. Molecular markers of risk of recurrence and poor prognosis were described previously from quantitative RT-PCR measurements of *DLGAP5* (*DLG7)*, *PINK1* and *BUB1B* expression [21]. Informed signed consent for the analysis of the tumor and for access to the data collected was obtained from all the patients, and the study was approved by the institutional review board of the Cochin Hospital.

**Table 1:** Clinical, histopathological, and molecular features of adrenal adenoma, IGF2-low carcinoma, and IGF2-high carcinoma.

	Adenomas (n = 87)	CS IGF2-low (n = 10)	CS IGF2-high (n = 43)	p-value (CS IGF2-low vs CS IGF2-high)
Sex (Men/Women)	8/79	2/8	10/33	1^a^
Age at diagnosis (median (range), year)	48 (22–76)	57.5 (26–81)	45 (15–79)	0.17^b^
Weight (median (range), g)	16 (5–240)	134.5 (40–551)	249 (50–2700)	0.09^ b^
Size (median (range), cm)	3.5 (2–10)	9 (4–16)	11 (5–24)	0.31^ b^
Weiss score (median (range))	0 (0–2)	6 (3–9)	6 (3–8)	0.7^ b^
ENSAT score (median (range))	1 (1–2)	2 (1–4)	2 (1–4)	0.52^ b^
Secretion (yes/no)	53/34	10/0	34/9	0.18^ a^
Ki67 (median (range), positive cells/1000)	0 (0–18)	14 (0–118)	8.5 (0–269)	0.85^ b^
Metastatic (yes/no)	0/87	5/5	22/20	1^ a^
Treatment (surgery/surgery + mitotane)	83/4	4/5	14/25	0.7^ a^
17p13 LOH (yes/no)	0/22	7/3	26/5	0.38^ a^
DLGAP5 (DLG7)-PINK1 elevated risk of recurrence (yes/no)	0/87	8/2	26/15	0.46^ a^
BUB1B-PINK1 poor prognosis (yes/no)	0/87	7/3	24/18	0.72^ a^
TP53 mutation (yes/no)	NA	3/4	5/33	0.09^ a^ *
Abnormal TP53 IHC (yes/no)	NA	4/4	8/27	0.19^ a^
CTNNB1 mutation (yes/no)	NA	2/6	6/32	0.61^ a^
Abnormal CTNNB1 IHC (yes/no)	NA	4/4	8/27	0.19^ a^

These various characteristics were determined as detailed in Material and Methods. *P*-values were calculated with the Fisher's exact test (^a^) or Wilcoxon test (^b^) and show no significant differences between IGF2-high and IGF2-low ACC. When numbers do not add up to 10 for IGF2-low and 43 for IGF2-high, this indicates that data are not available for all patients.

### Cellular models of *IGF2* knock-down in H295R cells

H295R cells, which are derived from an adrenal carcinoma overexpressing *IGF2*, were ordered from ATCC, and cultured as described previously [28].

For the transient knock-down of *IGF2*, siRNA duplexes against *IGF2* (forward sequence 5′UCGUUGAGGAGUGCUGUUUdTdT3′) and a control siRNA with no target in the human genome (forward sequence 5′GGCAUAGAUGUAGCUGUAAdTdT3′) were designed and obtained from Eurogentec. For the efficient knock-down of *IGF2*, 3.10^5^ cells were transfected twice with 50 pmol siRNA, at day 1 and 2 after cell plating, with the Effectene transfection reagent (Qiagen) according to manufacturer's instructions.

For the stable knock-down of *IGF2*, H295R cells stably expressing a Tet repressor (H295R/TR clone) were kindly provided by Dr Lalli [29]. A linker 5′GATCCCCATCGTTGAGGAGTGCTGTTTCAAGAGAACAGCACTCCTCAACGATGTTTTTA3′ 3′GGGTAGCAACTCCTCACGACAAAGTTCTCTTGTCGTGAGGAGTTGCTACAAAAATTCGA5′ was subcloned into the *BglII*-*HindIII* sites of the pSUPERIOR.puro vector (Oligoengine) to generate clones expressing a doxycycline-inducible IGF2 shRNA. H295R/TR cells were transfected further with the pSUPERIOR.puro/shRNA IGF2 plasmid and clones were selected with puromycin (5 µg/mL). A dose-response curve showed that 10 ng/mL of doxycycline was sufficient for the maximal knock-down of *IGF2* by the shRNA (data not shown).

Doxycycline does not modify *IGF2* expression in H295R/TR cells or control clones transfected with an empty pSUPERIOR.puro plasmid (data not shown).

### Cell Proliferation, apoptosis and cell cycle assays

Cell proliferation was measured with the CellTiter 96 wells Non-Radioactive Cell Proliferation Assay (Promega), according to manufacturer's instructions (quick protocol). Cells (8000/well) were plated on 96-well plates and the first measurement (D0) was made 3 days later. Optical absorbance was measured at 570 nm in a microplate reader.

Apoptosis was analyzed by staining cells with Annexin V with the Annexin-V-FLUOS Staining Kit (Roche), according to manufacturer's instructions. Cells were analyzed by flow cytometry with a Cytomics TM FC500 (Beckman Coulter), and data were processed with Cytomics RXP software.

Spontaneous apoptosis was studied in stable clones after 10 days of *IGF2* knock-down induced by doxycycline treatment.

TNF-alpha-induced apoptosis was analyzed in H295R cells after transient *IGF2* knock-down by siRNA for 48 h, followed by a 48 h treatment with 20 ng/mL TNF-alpha (eBioscience).

For cell cycle analysis, cells were seeded in 6-well plates. At time of analysis, cells were trypsinized, rinsed with PBS and fixed in 500 µL of 70% ethanol. They were then centrifuged, rinsed in PBS, and suspended in 50 µg/mL of propidium iodide + 100 µg/mL RNase A. Analysis by flow cytometry was performed with a Cytomics TM FC500 (Beckman Coulter), and data were processed with Cytomics RXP software.

### Quantitative RT-PCR

#### Reverse transcription of RNA from tumors

IGF2-high (23 samples) and IGF2-low (10 samples) ACC were studied by quantitative RT-PCR. Total RNA was extracted from tumors as described previously [21] and reverse transcribed (1.5 µg in 100 µl) with the High Capacity cDNA Reverse Transcription kit (Applied Biosystems).

#### Reverse transcription of RNA from cell lines

Total RNA was extracted from cell lines with the RNeasy Mini kit and RNase-free DNase Set (Qiagen) according to the manufacturer's instructions. Purified RNA was reverse transcribed (500 ng in 20 µl) with Superscript II RNA polymerase kit (Invitrogen).

#### RT-PCR

The expression of target genes was analyzed by quantitative PCR with a LightCycler Fast Start SYBR Green kit (Roche Diagnostics) according to the manufacturer's instructions. Briefly, 2.5 µl of 10-fold diluted reverse transcription product was used with 500 nM of each primer in a 10 µl reaction. Optimal annealing temperature, PCR efficiency, and PCR specificity (dissociation curve to verify amplification of one product) were determined with a four-point dilution curve. Conditions are described for all target genes in Table S1.

#### Analyses for tumors

Relative quantification of target cDNA was determined by calculating the difference in cross-threshold (*C*_T_) values after normalization to *18SRNA5* signals (ΔΔ*C*_T_ method), taking a normal adrenal sample as a reference. In our previous study, 18S mRNA was validated as the best reference gene, with perfect correlation between microarray and qPCR results [21]. Similar results were obtained using *GAPDH* as reference gene (data not shown). Each sample was analyzed in duplicate.

#### Analyses for cell lines

*IGF2* and *PP1A* mRNA levels were quantified using dilution curves included in the experiments. No variation of *PP1A* level was observed after siRNA or doxyxycline treatment, showing that it is a reliable reference gene for these experiments. Ratio between *IGF2* and *PP1A* levels was fixed to one when *IGF2* was not knocked-down (control siRNA or no doxycycline treatment).

### Western blot

IGF2-high and IGF2-low ACC (10 samples each) were studied by western blot. Whole cell protein lysates were obtained from tumors by extraction with RIPA lysis buffer (50 mM Tris–HCl, pH 7.5, 1 mM EDTA, 150 mM NaCl, 0.1% v/v Nonidet P-40 (NP40), 1 mM dithiothreitol (DTT)) with inhibitors of proteases (Complete Protease Inhibitor Cocktail Tablets, Roche) and phosphatases (PhosSTOP Phosphatase Inhibitor Cocktail Tablets, Roche). Western blotting was done according to standard procedures [30] with chemiluminescence and films (Amersham hyperfilm ECL, GE healthcare). Antibodies and conditions are described in Table S2.

Western blot bands were quantified with the GeneTools software (http://www.syngene.com/genetools-software-download).

### Transcriptomic analyses

Transcriptomic analyses of adrenocortical tumors (47 ACA, 23 IGF2-high ACC and six IGF2-low ACC) were reported previously [21]. All data are available at the ArrayExpress Web site (http://www.ebi.ac.uk/arrayexpress, experiment E-TABM-311) and were confirmed by quantitative RT-PCR of more than 100 genes.

Transcriptomic analyses of three different H295R clones, with or without *IGF2* knock-down for 2 or 10 days, was carried with Human gene 1.0 ST array (Affymetrix). Moreover, the same analysis was done after a 5 day transient inhibition of *IGF2* by siRNA in H295R cells, in normal (2% of Ultroser G serum, Pall Biosepra) or depleted medium (without UltroserG, depletion from one day before siRNA transfection to the end of the experiment). Total RNA was extracted from cell lines with the RNeasy Mini kit and RNase-free DNase Set (Qiagen) according to the manufacturer's instructions, and RNA quality was evaluated by Bioanalyzer (Agilent). All raw data for cell line microarray experiments are available upon request.

### 11p15 locus Methylation studies

The methylation of the telomeric and centromeric 11p15 Imprinting Center Regions, termed ICR1 and ICR2, respectively, were analyzed by allele-specific methylated multiplex real time quantitative PCR (ASMM RTQ-PCR) as described previously [31], [32]. These analyses were performed on 15 IGF2-high and nine IGF2-low tumors for which tumor DNA was available.

### Sequencing

The three coding exons and adjacent intronic regions of *IGF2* gene were amplified by PCR with the primers indicated in the lower part of table S1 and the amplicons were sequenced by the Sanger method (Sequencer Applied Biosystem 3130 XL ABI Prism 16 capillaries and Big Dye Terminator V1 Kit).

### Statistical analyses

Survival Kaplan-Meier curves, Fisher and Wilcoxon statistical tests were carried out with R software.

Genes differentially expressed between IGF2-high and IGF2-low ACCs in the transcriptomic study were determined with the limma *t*-test (R package).

Genes that showed significantly modified expression following *IGF2* knock-down in cellular models were defined with a paired *t*-test on the eight independent cellular experiments (three clones analyzed at 2 and 10 days after *IGF2* knock-down, and two siRNA experiments with 5 days of *IGF2* knock-down in normal or depleted medium, see above).

Pathways analysis was performed as follows: (i) Pathways were obtained from KEGG, Gene Ontology, Biocarta, MSigDB and Stanford Microarray Database websites. (ii) Gene lists tested for enrichment were based on the top 500, 250 and 100 probe sets sorted by *p*-value (for a given comparison – paired *t*-test in cell models and limma *t*-tests in tumors). These lists of up-regulated or down-regulated genes were analyzed for pathway enrichment. (iii) Hypergeometric tests were used to measure the association between gene lists and pathways.

## Results

### Tumor and Cell models

**Figure 1:**
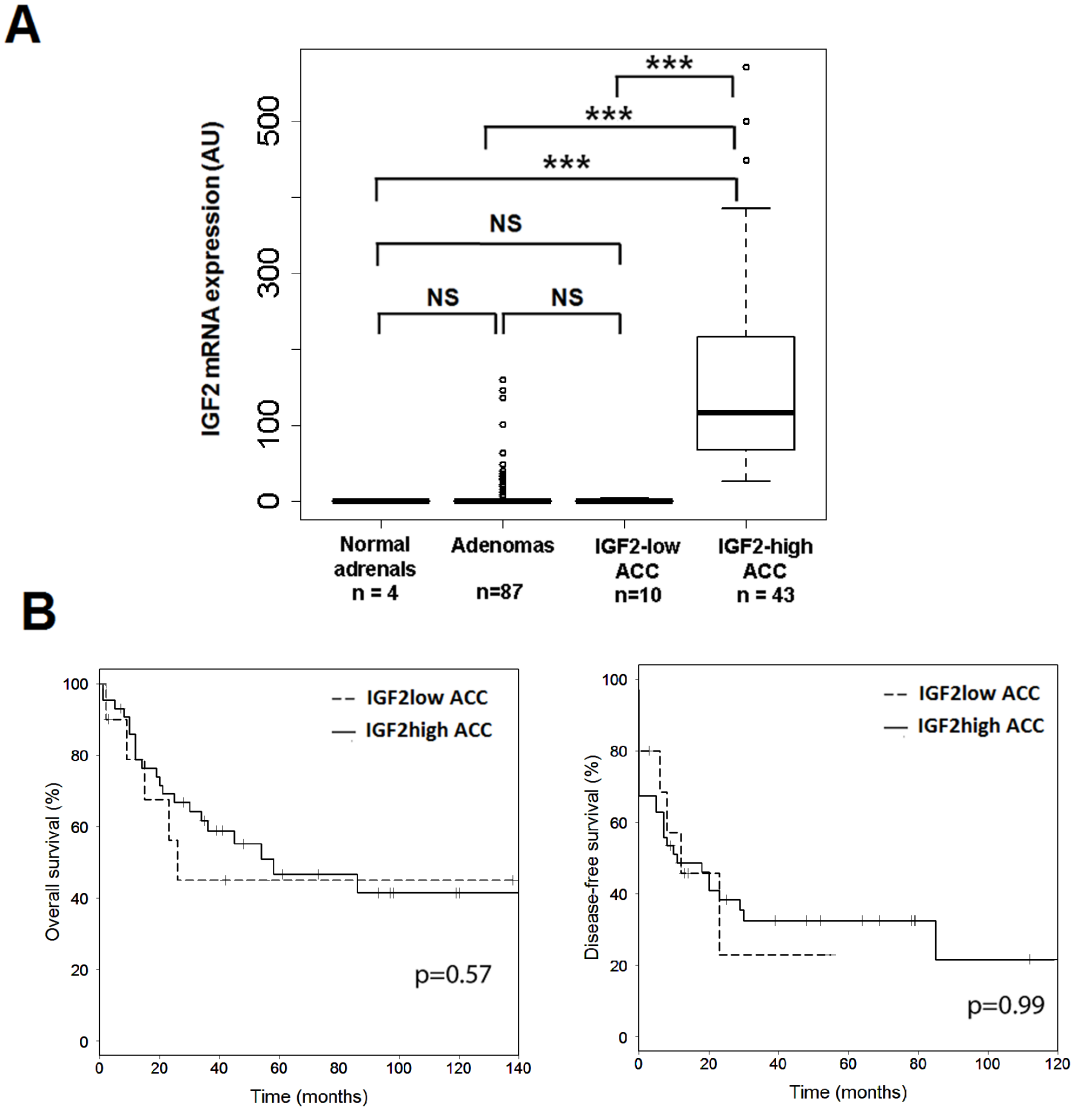
IGF2 mRNA levels and prognoses of IGF2-high and IGF2-low ACC. A: Boxplot showing IGF2 mRNA levels in normal adrenals (n = 4), ACA (n = 87), IGF2-low ACC (n = 10), and IGF2-high ACC (n = 43). IGF2-low ACC have similar levels of IGF2 to normal adrenals and ACAs. There is a minimal 6.5 fold difference between IGF2-high and IGF2-low ACC and there is a 200-fold difference between their median expressions. B: Kaplan-Meier survival curves showing overall (left) and disease-free (right) survival of patients with IGF2-low ACC (n = 10) or IGF2-high ACC (n = 43). *P*-values from a Cox regression analysis between IGF2-high and IGF2-low ACC are indicated, showing that there is no difference between the two groups.

We studied phenotypical differences between adrenocortical carcinoma cells with or without the overexpression of *IGF2* in two different models:

- Adrenocortical carcinoma samples with or without the overexpression of *IGF2*. The cohort included 53 patients who underwent surgery between 1993 and 2008 for an ACC. This cohort was divided into two groups on the basis of IGF2 mRNA abundance: the tumor was considered as IGF2-high when its expression was at least 25 higher than normal adrenals. IGF2-low ACC had levels similar to normal adrenals (0.025 to 4.07 fold) (Figure 1A).
- Adrenocortical carcinoma cells (H295R) express large amounts of IGF2. We knocked-down *IGF2* expression in these cells with either siRNA (transient knock-down) or with shRNA, which enables an inducible and stable knock-down of *IGF2*. *IGF2* expression was 84% lower than control values at days 2 and 3 after transient transfection (*p*<0.05) (Figure S1A) and remained significantly low (74% lower than control) at day 5 in three independent experiments. In four independent stably transfected clones treated with doxycycline, the abundance of IGF2 was significantly lower than in untreated clones (40 to 74% lower at day 2 and 64 to 89% lower at day 10) (Figure S1B).

### IGF2-high and IGF2-low adrenocortical carcinoma have similar clinical, histopathological and molecular markers and features

The first goal of this study was to compare the phenotypic traits of ACC that overexpress *IGF2* with those that do not. We analyzed first the clinical differences between IGF2-high and IGF2-low ACC. As indicated in Table 1, sex ratio, age at diagnosis and size and weight of tumors were not strikingly different between the two types of tumors. In addition, proliferation and invasiveness were not significantly different as assessed by the Weiss score, Ki67 index and ENSAT stage [33]. The occurrence of metastasis was also similar. Patients received similar treatment regimens (surgery alone or surgery + mitotane) and molecular markers of carcinoma diagnosis and prognosis were not different in the two groups. These observations were supported by the comparison of the overall and event-free survival for IGF2-high carcinoma and IGF2-low carcinoma (Figure 1B). Again, the two groups of ACC presented similar survival (Logrank p = 0.57 and p = 0.99 for overall and event-free survival, respectively), which was significantly different from the survival of patients with ACA (p<1.10^-14^ for both overall and event-free survival, data not shown). In the previously described unsupervised clustering of the ACC transcriptome, which identified two clusters with very different prognoses (Figure 1A of reference [21]), IGF2-low ACC did not cluster apart from IGF2-high ACC (data not shown). Altogether, these data indicate that there is no major phenotypic difference between the IGF2-low and IGF2-high carcinoma in terms of clinical, histological and molecular markers. IGF2 status is clearly correlated with malignancy [14], but it is not a prognostic marker in carcinoma.

We used the same transcriptomic data [21] to identify genes differentially expressed between IGF2-high and IGF2-low ACC and we searched for pathways enriched in differentially expressed genes (Table S3-ACC-pathways). Gene expression, transcription, cell cycle regulation (especially the G1/S transition), and p53 were among the pathways enriched in genes highly expressed in IGF2-high tumors. Multidrug resistance factors, Jak-STAT, and response to oxidative stress were among the pathways enriched in genes highly expressed in IGF2-low tumors.

We carried out the same pathway enrichment analysis on the transcriptomic data of H295 cells with or without the knock-down of *IGF2* (Table S4). The expression of genes involved in the G1/S transition of cell cycle, apoptosis, insulin and p53 signaling was significantly impaired by *IGF2* knock-down. Genes up or down-regulated both in tumor and cellular models, which are potential targets of IGF2, are listed in Table S5.

### Expression and activity of IGF signaling pathways in IGF2-high and IGF2-low tumors and cells

**Figure 2:**
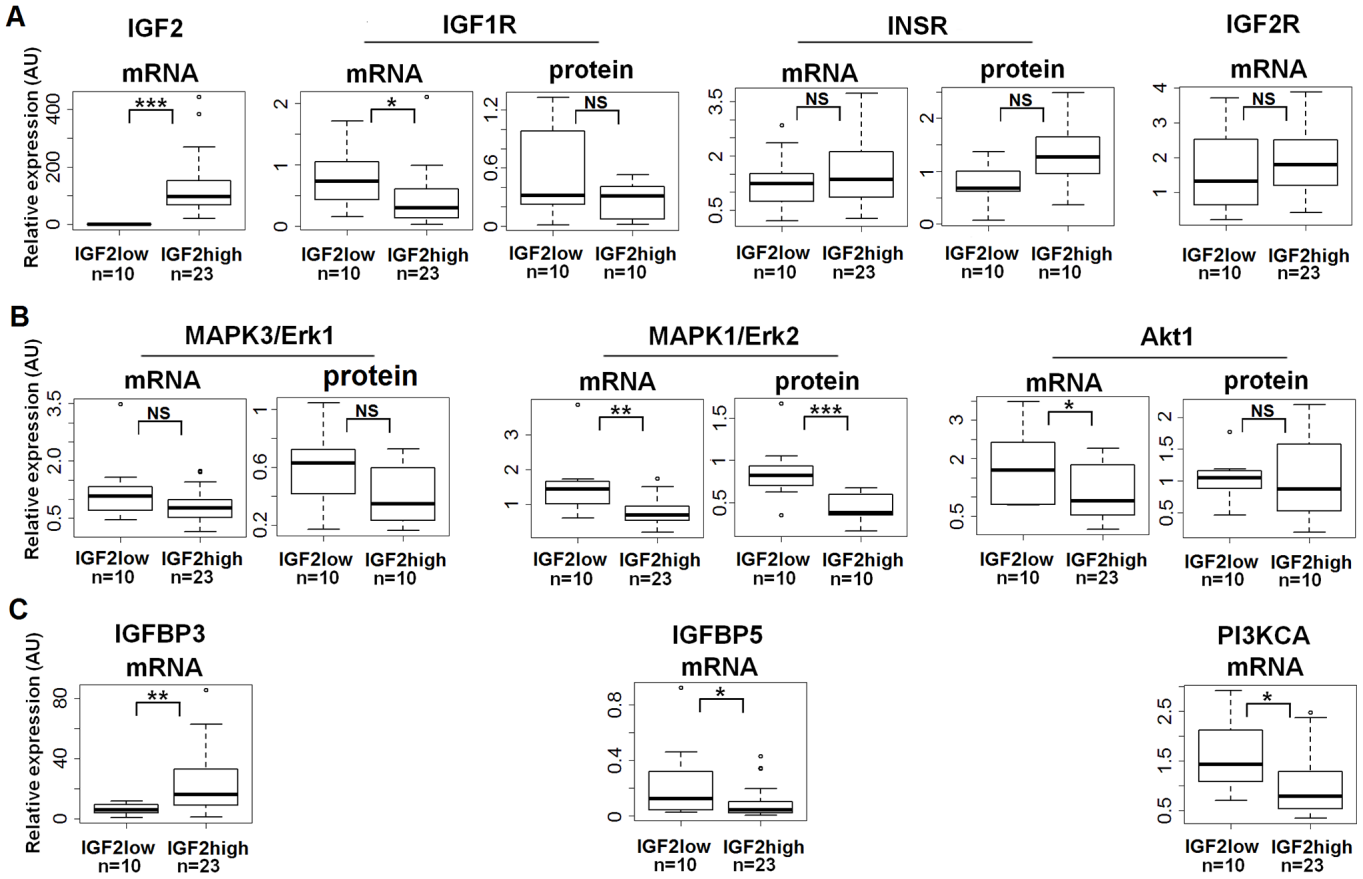
Quantitative expression of IGF signaling pathway members. Boxplots showing the mRNA (quantitative RT-PCR) and/or protein (western blot) levels of several IGF2 pathway members in IGF2-high (n = 23 for RT-PCR and n = 10 for western blot) and IGF2-low (n = 10) ACC. Y-axis for mRNA: result of the ΔΔ*C*_T_ method (2^-ΔΔ*C*T^ value), with *RNA18S5* as a reference gene. Y-axis for protein: results of the quantification of the western blot bands, normalized to actin. Wilcoxon test results (*p<0.05; **p<0.01; ***p<0.00; NS = not significant) are indicated for each mRNA or protein studied. A: IGF2 and its receptors. B: Erk and Akt. C: other IGF2 pathway members with significantly different expression between IGF2-high and IGF2-low ACC.

Data from microarray experiments analyzing the transcriptome of adrenocortical tumors [21] suggest few differences in the expression of IGF pathway members between IGF2-low and IGF2-high carcinoma. These results were obtained with a limited number of IGF2-low ACC (n = 6); therefore, we sought to validate these findings. We assessed the expression of 18 partners associated with the IGF signaling pathway, including receptors, IGFBPs, Akt, PI3K and Erk isoforms, by quantitative RT-PCR in 10 IGF2-low and the 23 IGF2-high carcinoma. In addition, we analyzed the protein abundance of receptors, Akt and Erk isoforms by western blotting in 10 tumors of each group. Most of the 18 studied genes were not differentially expressed between IGF2-high and IGF2-low ACC, and the few significant differences were modest (Figure 2A). There was a strong correlation between transcriptomic and quantitative RT-PCR data, and we confirmed several of these results by western blotting. The most important findings of this study are presented in Figure 2 and the complete data set is accessible in Table S6. Notably, the expression of the short isoform of INSR was not different between the two groups (Table S6). Significant differences included a 2 to 3-fold higher abundance of Erk2 protein and mRNA, total Erk (Erk1 + Erk2) proteins, IGFBP5 and PI3KCA mRNAs in the IGF2-low tumors (Figure 2B) and a 3-fold higher abundance of mRNAs encoding IGFBP3 in the IGF2-high tumors (Figure 2C). We confirmed these findings in the cellular model; the efficient knock-down of *IGF2* did not modify the expression of the factors assessed by transcriptomic analysis (Table S3-cellular models-genes) and by western blotting (Figure S2 B, C, E, G). These results indicate that overexpression of *IGF2* has no major effect on the expression of the members of its signaling pathway.

**Figure 3:**
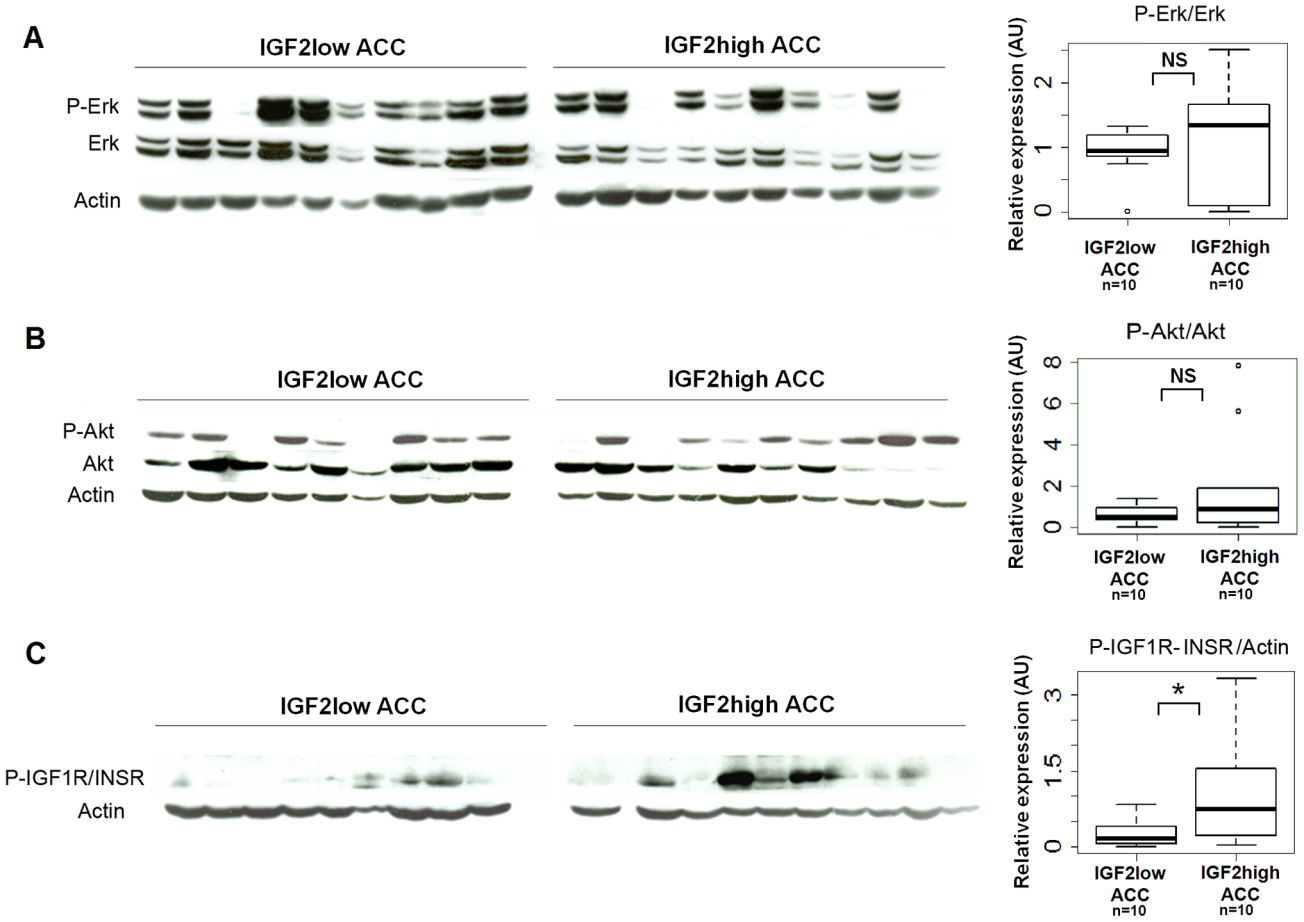
IGF signaling pathway activation in IGF2-high and IGF2-low ACC. The activation of Erk (A), Akt (B), and IGF1R/INSR receptors (C) was analyzed by western blotting with antibodies directed against the phosphorylated form of these proteins, in IGF2-high (n = 10) and IGF2-low (n = 10) ACC. Boxplots show the quantification of the results of western blots. Y-axis: results of the quantification of the western blot bands normalized to total Erk, Akt or actin. Wilcoxon test results (*p<0.05; NS = not significant) are indicated. The phosphorylation of the receptors is higher in IGF2-high than in IGF2-low ACC, whereas the activation of Erk and Akt downstream pathways is similar.

We then examined whether the activation of the IGF, Erk, and Akt pathways was different between IGF2-high and IGF2-low ACC. We assessed the abundance of the phosphorylated forms of these proteins in 10 tumors from each group by western blotting. The phosphorylation of IGF1 and insulin receptors was higher in IGF2-high carcinoma than in IGF2-low carcinoma, but there was no difference in the phosphorylation status of Akt and Erk1/2 when normalizing to total Akt or Erk1/2 respectively (Figure 3) or actin (data not shown). These results are very surprising because the higher expression of IGF2 in IGF2-high ACC should result in a robust stimulation of these two pathways. We cannot exclude that the residual expression of IGF2 in IGF2-low ACC may stimulate these pathways, although this is unlikely. Another explanation is that other growth factors or their receptors are more expressed in IGF2-low tumors than in IGF2-high tumors. Indeed, our comparison of the transcriptome between IGF2-high and IGF2-low ACC identified some growth factors (*FGF9*, *PDGFA*, *TNFSF10*, and *TNFSF4*) that were 2-fold more expressed in IGF2-low ACC than in IGF2-high ACC (underlined in yellow in Table S3_ACC_genes). Differential expressions of *FGF9* and *PDGFA* were confirmed by quantitative RT-PCR (Table S6).

We carried out similar experiments in H295R clones. INSR/IGF1R phosphorylation was unaffected by *IGF2* knock-down (Figure S2A). Moreover, Erk and Akt pathways (Figure S2D-G) were not inhibited. Surprisingly, we observed a significant stimulation of Erk1/2 phosphorylation after 7 and 10 days of *IGF2* knock-down. These results prompted us to analyze the tumorigenic role of IGF2 in adrenocortical cells.

### *IGF2* knock-down in H295R cells impairs proliferation, leads to a G1 arrest and promotes apoptosis

**Figure 4:**
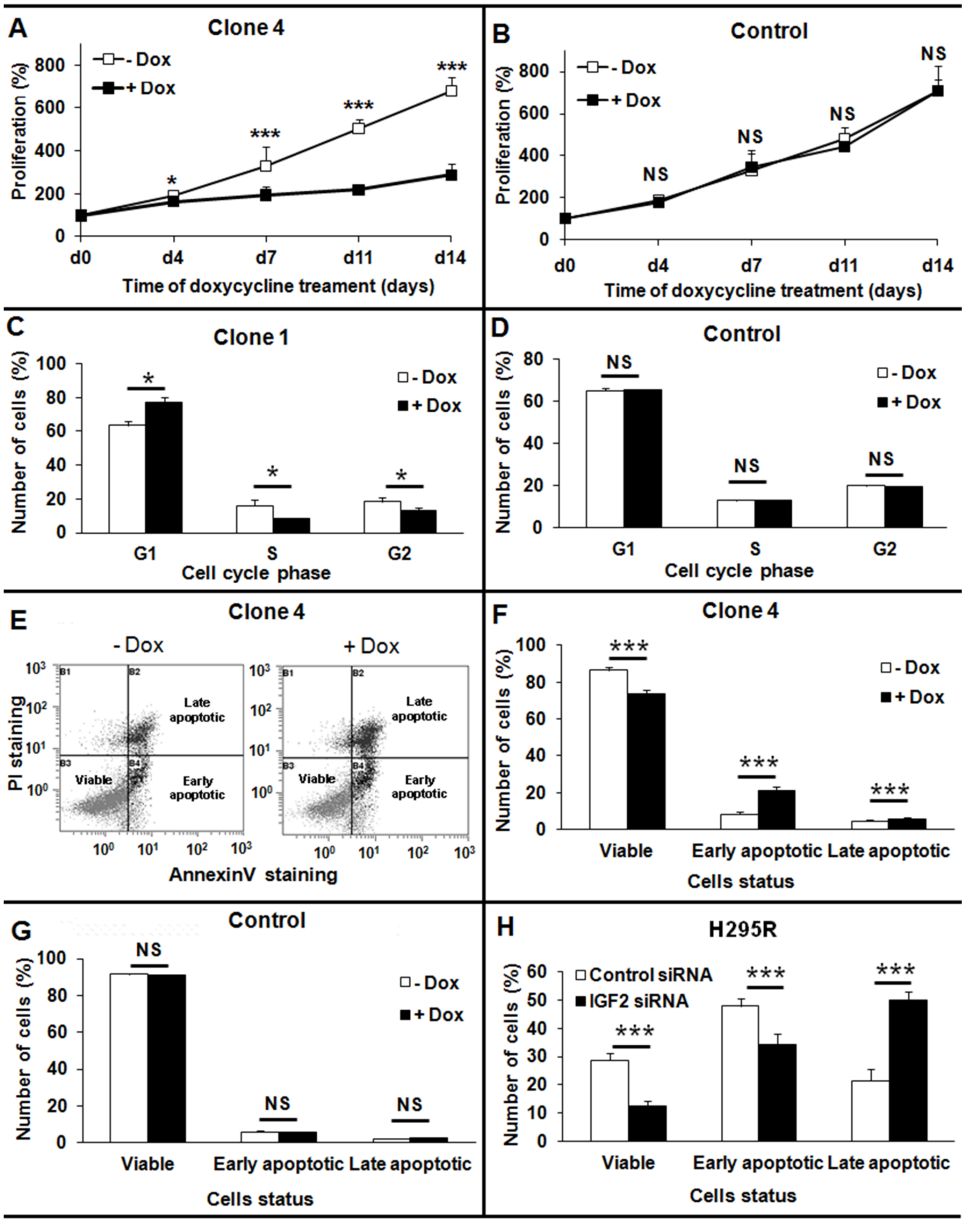
Consequences of *IGF2* knock-down on cellular growth, apoptosis, and the cell cycle in H295R cells. A, B: Consequences of long-term knock-down of *IGF2* on H295R cell growth as assessed by a MTT proliferation test. A: Doxycycline treatment leads to *IGF2* knock-down and to a significant impairment of cellular proliferation in a clone with integrated IGF2 shRNA. Results are presented for clone 4, which showed the most significant impairment of cell growth B: Doxycycline (black square) does not inhibit proliferation in a control clone. C, D: Effect of *IGF2* knock-down on the cell cycle. After 7 days of doxycycline treatment, the cell cycle was analyzed by PI staining and flow cytometry. Cells expressing IGF2 shRNA were largely arrested in the G1 phase of the cell-cycle and few cells were in S phase (C). Results are presented for clone 1, which showed the most significant G1 cell cycle arrest. Doxycycline treatment did not affect the cell cycle of a control clone (D). E-H: Apoptosis studied by flow cytometry after PI (Propidium iodide) and Annexin V staining. E: Example of FACS results for clone 4, showing the apoptotic status of cells according to Annexin V (X axis) and PI (Y axis) staining. F, G: Study of non-induced apoptosis after prolonged *IGF2* knock-down. Ten days after shRNA induction by doxycycline treatment, cells were stained and analyzed by flow cytometry. Doxycycline treatment did not affect apoptosis of the control clone without integrated shRNA (G). Both early and late apoptosis were significantly higher in cells expressing IGF2 shRNA than in control cells (F). Results are presented for clone 4, which showed the most significant difference in apoptosis. H: Study of TNF-alpha-induced apoptosis after the transient knock-down of *IGF2* in H295R cells. Cells were treated with TNF-alpha 48 h after siRNA transfection, and were analyzed by flow cytometry 48 h later. The number of viable cells was significantly lower in cells transfected with siRNA against IGF2 (black bars), and the percentage of cells in late apoptosis was significantly higher than in cells transfected with a control siRNA (white bars). Results were analyzed with the Wilcoxon test. *: *p*<0.05. ***: *p*<0.001. All the results presented in this figure are representative of at least three independent experiments.

The stable knock-down of *IGF2* dramatically impaired cell proliferation in an MTT assay (Figure 4A), whereas cell proliferation was not affected in a doxycycline-treated control clone (Figure 4B). This effect was apparent as soon as 4 days after the initiation of doxycycline treatment, and was maximal at D14. At this time point, the number of cells of clone 4 treated with doxycycline was 2.4 times lower than the number of cells not treated with doxycycline (676±6.7 without Dox versus 288±13 with Dox, *p*<0.001). We observed a similar impairment of cell proliferation in the other three clones, ranging from 1.7 to 2.3-fold reduction of proliferation at D14 (data not shown). These data indicate an important role for IGF2 in the growth of the adrenocortical tumor cell line H295R.

We then investigated the consequences of *IGF2* knock-down on the cell cycle. A significant proportion of cells were arrested in G1 after 7 days of doxycycline treatment (Figure 4C). For clone 1, the percentage of cells in G1 was 77.3%±3.1 with doxycycline versus 63.5%±2.4 without (*p*<0.05). In parallel, the number of cells in S phase was 1.95-fold lower in doxycycline-treated cells (8.2%±0.7 versus 15.9%±3.6). We confirmed these findings in the other three clones; in doxycycline-treated cells the percentage of cells in G1 was 5 to 14% higher and the percentage of cells in S phase was 11 to 28% lower than in control conditions (data not shown). The cell cycle was unaffected in a control clone treated with doxycycline (Figure 4D). Microarray experiments showed down-regulation of two cyclin-dependant kinases (*CDK2* and *CDK8*) and one of their positive regulators (*CKS1B*) (Table S4). These data support a role for IGF2 in the G1/S transition in adrenocortical cell line H295R.

We also studied the effect of *IGF2* knock-down on apoptosis by FACS (Figure 4E). We analyzed spontaneous apoptosis in stable clones after 10 days of doxycycline treatment to induce *IGF2* knock-down. Both early and late apoptosis were significantly higher in cells expressing IGF2 shRNA than in control cells (Figure 4F). For clone 4, the percentage of early apoptotic cells was 20.8%±2.4 with doxycycline and was 8.4%±1.3 without doxycycline (2.5 fold, *p*<0.001). The percentage of late apoptotic cells was 5.5%±0.9 with doxycycline and was 4.4%±0.7 without doxycycline (1.3 fold, *p*<0.001). We confirmed this high rate of apoptosis in the other three clones; the number of apoptotic cells was 1.8 to 1.9 fold higher in doxycycline-treated cells than in control cells (data not shown). Apoptosis was unaffected in a control clone treated with doxycycline (Figure 4G).

We also transfected H295R with siRNA against *IGF2* and induced apoptosis in these cells by TNF-alpha 48 h after transfection (Figure 4H). These transient knock-down experiments confirmed the effect of *IGF2* knock-down on apoptosis and demonstrate further the anti-apoptotic role of IGF2 in the adrenocortical tumor cell line H295R.

Transcriptome analysis shows an increase in *PMAIP1* and *BCL2L11* expression following IGF2 knock-down suggesting the involvement of the intrinsic apoptotic pathway. Two apoptosis inhibitors (*CDKN1A* and *RNF7*) are also down-regulated (Table S4).

### 11p15 methylation profiles differ between IGF2-high and IGF2-low ACC

Finally, we investigated for the possibility of an additional genetic or epigenetic event at the 11p15 locus, which can explain the low *IGF2* expression in the IGF2-low ACC.

We first looked for genetic alterations in the *IGF2* gene by direct sequencing of the 3 coding exons in six IGF2-low ACC for which DNA was available. No deleterious point mutation was detected in the six samples (data not shown).

**Figure 5:**
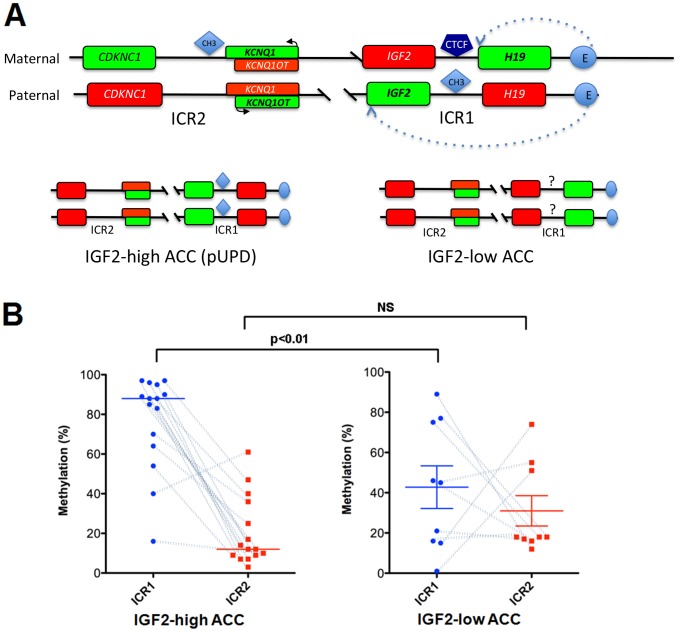
Structure and methylation of the 11p15 locus. A. Schematic representation of the 11p15 locus. The 11p15 locus is represented with the 2 differentially imprinted regions (ICR1 & ICR2). In the maternal allele *H19* is expressed but not *IGF2* as the consequence of CTCF binding to a sequence located between the 2 genes and acting as an insulator. Therefore the enhancer (E) can only activate the transcription of the most proximal gene. The methylation (CH3) of this sequence (paternal allele) prevents the binding of CTCF, allows the expression of *IGF2* and represses that of *H19*. The methylation (maternal allele) or not (paternal allele) of ICR2 results in the opposite expression of *CDKN1C*, *KCNQ1* and *KCNQ10T*. Genes with activation or repression of their expression are indicated in green or red respectively. The most frequent patterns observed in IGF2-high (left) and IGF2-low (right) ACC are indicated in the lower part of the figure. B: Methylation of the 11p15 imprinting center regions in IGF2-high (n = 15) and IGF2-low (n = 9) ACC. The percentage of methylation (y-axis) is shown for ICR1 (blue circles) and ICR2 (red squares). Wilcoxon test results (**p<0.01; NS = not significant) are indicated. Dotted lines connect data from the same tumor, and indicate paternal uniparental disomy (pUPD) when ICR1 is highly methylated and ICR2 is weakly methylated. This pUPD pattern is present in almost all IGF2-high ACC, whereas it is present in only three out of nine IGF2-low ACC.

**Figure 6:**
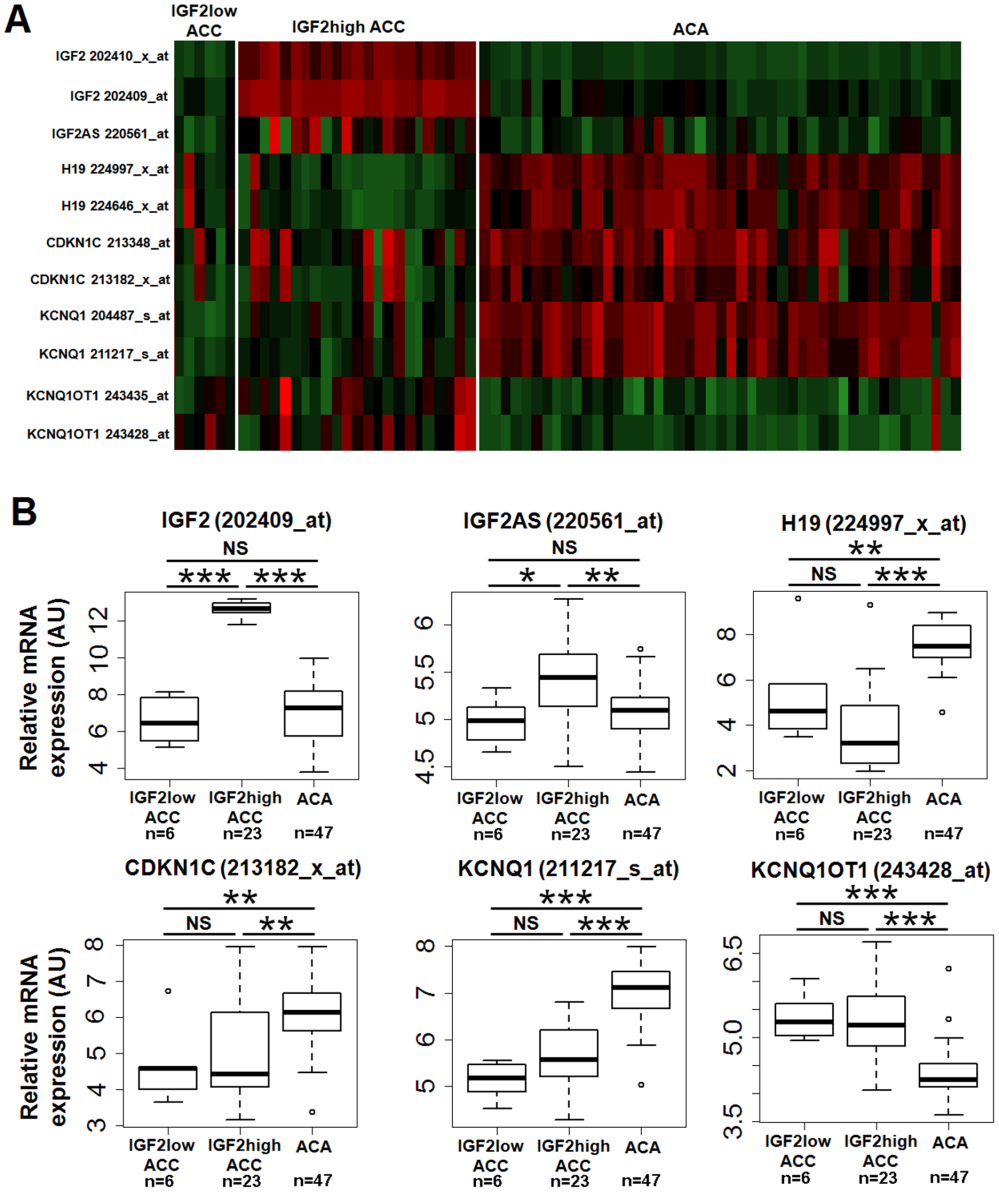
Expression of the genes in the 11p15 imprinted region. A: Snapshot heat map of the 11p15 chromosomal region generated from a previous transcriptomic study (21). Expression levels of imprinted genes *IGF2*, *IGF2AS*, *H19*, *CDKN1C*, *KCNQ1*, and *KCNQ1OT1* are shown for 47 ACA, six IGF2-low ACC and 23 IGF2-high ACC. Red: high expression. Green: low expression. B: Boxplots showing expression data for the same genes. Wilcoxon test results (*p<0.05; **p<0.01; ***p<0.001; NS = not significant) are indicated. Except for *IGF2* and *IGF2AS*, there are no significant differences between IGF2-high and IGF2-low ACC.

We next analyzed the methylation of the 11p15 locus. Briefly, two Imprinting Center Regions (ICR1 and ICR2) are either methylated or unmethylated in the 11p15 locus. Methylation of ICR1 controls the expression of the *IGF2/H19* genes and its methylation prevents the binding of the CTCF protein to DNA, which acts as an insulator between the two genes (Figure 5A). Therefore, on the paternal allele where ICR1 is methylated, *IGF2* is expressed and *H19* is not. ICR1 is not methylated on the maternal allele, thus *H19* is expressed and *IGF2* is not. Similarly, ICR2 is methylated on the maternal allele resulting in the expression of *CDKN1C* and *KCNQ1* but not *KCNQ10T1*, whereas ICR2 is not methylated on the paternal allele resulting in the expression of *KCNQ10T1* but not *CDKN1C* and *KCNQ1*. The mechanism of *IGF2* expression in adrenocortical tumors has been extensively analyzed, and it is now clear that this overexpression is due, at least in part, to paternal uniparental disomy (pUPD) at the 11p15 locus [14], [34]. This pUPD results in an overexpression of *IGF2*, but also of *KCNQ10T1*, whereas the expression of *H19*, *CDKN1C* and *KCNQ1* is impaired. In the present cohort, UPD was characterized previously in 20 IGF2-high and five IGF2-low ACC by Southern blotting [15]. Of these samples, 90% of IGF2-high ACC showed pUPD and all IGF2-low ACC showed the same alteration. We looked at the expression of the five imprinted genes in the previous transcriptomic study [21] to confirm these results. As shown in Figure 6 A and B, the expression pattern of these five genes is compatible with pUPD in IGF2-high tumors. In IGF2-low tumors, the expression of *CDKN1C*, *KCNQ1* and *KCNQ1OT1* is indicative of the expected demethylation of ICR2; however, the low expression of *IGF2* and the moderate expression of H19 suggest an unexpected impairment to methylation at ICR1, at least in some tumors.

We confirmed this hypothesis by the analysis of methylation at ICR1 and ICR2 in 15 IGF2-high and nine IGF2-low tumors (Figure 5B). This analysis showed that 80% of IGF2-high tumors had a methylation profile compatible with pUPD, whereas only 30% of IGF2-low tumors had such a pattern. Six of the nine IGF2-low tumors had low levels of methylation at ICR1, in accordance with the absence of *IGF2* expression. Therefore, these observations suggest that differences in ICR1 methylation explain the difference in *IGF2* expression between the two groups of tumors.

## Discussion

The role of IGF2 in the progression of adrenocortical carcinoma (ACC) has been debated for almost two decades. This question is becoming increasingly important to address, and may help the design of targeted therapies for this aggressive tumor which has a very poor prognosis. Two observations implicate IGF2 in ACC tumorigenesis: (i) the antiproliferative consequences of the inhibition of IGF2 function in ACC cell lines, and (ii) the overexpression of *IGF2* in ACC.

In the present work, we investigated the role of IGF2 in ACC by several original approaches including phenotypic comparison between IGF2-high and IGF2-low ACC carcinoma, transcriptomic analysis, and knock-down of *IGF2* in H295R cells with siRNA.

Logié *et al.* were the first to report that antibodies against IGF2 and IGF1R inhibit the proliferation of H295R cells [16]. More recently, it has been demonstrated that an anti-IGFR1 monoclonal antibody (IMC-A12) or a tyrosine kinase inhibitor specific for IGF1R (NVP-AEW541) are able to inhibit the proliferation of H295R cells both *in vitro* and in mouse xenografts [18]. This growth-promoting effect has also been demonstrated in other adrenocortical tumor cell lines: SW-13 cells do not express high levels of IGF2 and mouse Y1 cells do not express IGF1R; however, SW-13 cells proliferate rapidly in the presence of IGF2 [35] and Y1 cells proliferate rapidly when IGF1R is overexpressed [36]. We used siRNA to inhibit the endogenous production of IGF2, which demonstrated the important role of IGF2 in proliferation, cell cycle progression and apoptosis of H295R cells. These results confirm that IGF2 is an important growth factor at least *in vitro* in the adrenocortical cell line H295R.

Mouse models in which IGF2 is specifically overexpressed in the adrenal cortex have been recently developed [24], [25]. In the first of these models, the overexpression of *IGF2* did not initiate adrenal tumorigenesis. Its overexpression in mice expressing a constitutively active form of beta-catenin in the adrenal cortex resulted in adrenal hyperplasia, but did not modify tumor phenotype [24]. In another mouse model of Wnt/beta-catenin pathway activation in the adrenal cortex, (the adrenocortical *APC* KO mouse), Heaton *et al.* were also unable to identify any effect of *IGF2* overexpression on tumor progression [25]. This may be due to differences between species; nonetheless, these concordant observations introduce some doubt on the role of IGF2 in the initiation/progression of adrenocortical tumorigenesis.

*IGF2* is expressed in the normal adrenal cortex and ACA, but accumulating data demonstrate that *IGF2* is the most differentially expressed gene between malignant and benign adrenocortical tumors [14], [15], [19]-[22], [34]. In these studies, the abundance of IGF2 mRNA was 14 to 120-fold higher in ACC than in ACA, depending on the probe used. Similarly, overproduction of the IGF2 protein and its various isoforms has been demonstrated (x8 to 80), with a strong correlation between protein and mRNA abundance [13]. In the present cohort, we found that the expression of *IGF2* was 20-fold higher in ACC than in ACA, which confirms that *IGF2* is the most differentially expressed gene between malignant and benign tumors [21].

We explored whether this difference in *IGF2* expression was associated with differences in the phenotype or tumor biology between the two groups of ACC. The clinical presentation of IGF2-high and IGF2-low tumors was the same. Therefore, it is not surprising that the overall and disease free survival did not differ between patients with IGF2-high and patients with IGF2-low tumors. This result is in agreement with previous reports indicating that *IGF2* overexpression has no predictive value for prognosis or metastasis [19]. This inability to distinguish the two groups of tumors based on clinical characteristics was associated with similar proliferative histological features as assessed by comparable median Weiss scores and Ki67 indexes. In addition, classical molecular markers of adrenocortical carcinoma could not distinguish between IGF2-high and IGF2-low tumors, and although there were some differences in the transcriptome profiles between these two groups of tumors in the supervised analysis, there was no obvious difference in the unsupervised analysis [21]. Altogether, these data indicate that the major difference between the two groups of tumors is *IGF2* expression.

We next analyzed whether *IGF2* overexpression was associated with changes in the expression or activity of members of the IGF signaling pathway. Several reports have shown differences in the abundance of *IGF1R* [19], [37]-[40] *IGF2R* [41] and *IGFBP2* protein but not mRNA [13] between ACC and ACA. In addition, there are conflicting reports about whether the activity of the Akt/PI3kinase pathway is higher in malignant tumors than in benign tumors [18], [42]. In the microarray data of our series of 140 adrenocortical tumors, the expression of *IGF1R* in ACC was similar to that in ACA (data not shown). The expression of *IGF2R* was moderately but significantly higher in ACC than in ACA, in contrast with the expected LOH of this gene in ACC, which codes for an inhibitor of proliferation [41]. In addition, these data confirm *IGFBP2* is not differentially expressed between ACA and ACC, but indicate several significant differences in the expression of *IGFBP*, including the higher expression of (*IGFBP3*) and the lower expression of (*IGFBP5* and 6) in ACC than in ACA.

We also compared the expression and activity of the partners of the IGF signaling pathway between IGF2-high and IGF2-low ACC. Surprisingly, none of these genes showed any difference in expression between the two types of tumor both at the mRNA and protein levels, except for Erk2. This result contrasts with the literature, which indicates that *IGF1R* overexpression is a feature of many cancers [43] and that MEF cells with LOI at 11p15 (the classic mechanism for *IGF2* overexpression) express more IGF1R and INSR than cells without LOI [44].

In addition, *IGF2* expression did not affect significantly Akt and Erk phosphorylation, and therefore the activity of the tyrosine kinase signaling pathways, although the activation of IGF1R/INSR was significantly higher in IGF2-high ACC than in IGF2-low ACC. Similarly, the knock-down of *IGF2* in H295 cells inhibited cell proliferation and stimulated apoptosis without any identifiable change of PI3K/Akt and MAP kinase signaling pathway activities. This may be due to the transitory nature of this inhibition, which is rapidly compensated either by IGF1R/INSR desensitization or by activation of other growth promoting pathways. The most probable explanation for these discrepancies is that many other growth factors that signal through tyrosine kinase receptors are active in ACC. Several other growth factor receptors (*FGFR1, FGFR4* and *EGFR*) are overexpressed in ACC [38], [45]. The comparison of the transcriptome between IGF2-high and IGF2-low ACC also showed that the expression of some growth factors (FGF9, PDGFA) was higher in IGF2-low ACC than in IGF2-high ACC. Altogether, these data suggest that many other growth factors or alterations are involved in ACC progression.

Finally, we explored the molecular mechanism, which may explain differences in *IGF2* expression amongst ACC. The *IGF2* gene lies on an imprinted region of chromosome 11p15, which is a region with a complex epigenetic regulation. The molecular mechanism of *IGF2* overexpression in adrenocortical tumors is linked with paternal UPD (see the results section for details), resulting in methylation of ICR1 and demethylation of ICR2 [14], [34]. We identified pUPD in most IGF2-high ACC of our series; these samples showed the expected methylation profiles at ICR1 and ICR2 (80% of the tumors) and the expression of the five imprinted genes at this loci differed as expected from their expression in ACA. This pUPD is considered as an early event in the tumorigenesis process because it is absent in most adenoma (90%) and is present in most carcinoma (80 to 90% depending on the series, 82% in our series). In IGF2-low tumors, we found similar pUPD and hypomethylation of ICR2 with corresponding modifications of imprinted gene expression; interestingly however, most of these tumors also showed low methylation of ICR1 associated with a low expression of *IGF2* and a moderate expression of *H19*. This additional epigenetic event may explain the low production of IGF2 in IGF2-low tumors.

In conclusion, most ACC express large amounts of IGF2, which appears to be a driving force for the progression of tumorigenesis. This hypothesis is being tested in ongoing trials involving anti-IGF therapies [46]. *IGF2* is not overexpressed in a small subset of ACC, as a result of epigenetic modifications at the 11p15 locus. The origin of this subset of tumors is unclear. IGF2-high and IGF2-low tumors present no major clinical and transcriptomic differences and both show pUPD, suggesting a shared mechanism of tumorigenesis. It is not known whether *IGF2* overexpression is absent at the beginning of tumorigenesis or whether it is lost during the progression of the IGF2-low tumor. However, it is probable that in this small subset of IGF2-low ACC other growth factors and signaling pathways compensate for low *IGF2* expression, which creates opportunities for the design of other therapies targeting these factors.

## Supporting Information

Figure S1 **Cellular models of IGF2 knock-down in adrenal carcinoma.** A: Transient extinction of *IGF2* expression by siRNA in H295R cells. Cells were transfected twice at day 1 and 2 after plating, either with siRNA against IGF2 (black bars) or with control siRNA (white bars). Levels of IGF2 mRNA were measured by qRT-PCR at days 2, 3, and 5 after the first transfection. *IGF2* expression was reduced by 85% at days 2 and 3, and 74% at day 5. B: Long-term extinction of *IGF2* by shRNA in H295R cells. Levels of IGF2 mRNA were measured by qRT-PCR after 2 (d2) or 10 (d10) days after doxycycline treatment (black bars), and compared with those obtained in the absence of doxycycline (white bars). Results are presented here for 4 different clones, with 40 to 75% (d2) or 65 to 90% (d10) reduction of IGF2 expression. *PP1A* gene is used as a reference in both experiments and expression ratio between IGF2 and cyclophilin has been considered as 1 for the control experiment (control siRNA for A and no doxycyclin for B). Results were analysed using Wilcoxon test. *: p-value<0.05 Results are representative of at least 3 independent experiments.
(TIF)

Figure S2 **Study of IGF2 receptors, Erk, and Akt activation by western blot** **after 2, 7, or 10 days of IGF2 knock-down in stably transfected clones.** For each clone, expression of proteins was normalized to GAPDH expression, and fixed to 1 in the absence of doxycycline (white bars). Means and standard deviations of these ratios for 3 different clones after doxycycline treatment are indicated (black bars). A, D, F: Activated proteins as determined by their level of phosphorylation. B, C, E,G: Total proteins A, B, C: IGF2 receptors. D,E: Erk. F,G: Akt. Results were analysed using Wilcoxon test. *: p-value<0.05.
(TIF)

Table S1 **Primers for quantitative RT-PCR and sequencing.** Sequences, annealing temperatures, and PCR efficiencies are presented for each quantitative RT-PCR primer set.
(XLS)

Table S2 **Western blot antibodies.** Reference, supplier and dilution are indicated for the different antibodies used in this study.
(XLS)

Table S3 **Results of transcriptomic studies on tumors.** ACC-genes worksheet bar: from the transcriptome study already published (21), genes with significantly different expression between IGF2-low and IGF2-high ACC were determined using Limma T-Test. Green: genes significantly more expressed in IGF2-high ACC. Red: genes less expressed in IGF2-high ACC. A list of the genes without significantly different expression is available upon request. GM: Geometric Mean (log2). FC: Fold Change (log2). ACC-pathways worksheet bar: Pathways with enrichment in IGF2-high or IGF2-low ACC. Green: genes significantly more expressed in IGF2-high ACC. Red: genes less expressed in IGF2-high ACC. In the “Summary” column, enrichment of the pathway with genes up or down regulated in IGF2-low ACC is shown. A list of the pathways without enrichment in IGF-high or IGF2-low ACC is available upon request.
(XLS)

Table S4 **Transcriptomic studies on cellular models.** Cellular models-genes worksheet bar: ratios of gene expression with and without *IGF2* knock-down in different experiments with cellular models. Log2r:Log2 of the ratio. si.serum and si.noserum: experiments of 5-day *IGF2* extinction with IGF2 siRNA with complete or depleted culture medium, respectively. Experiments with 2 (d2) or 10 (d10) days of *IGF2* knock-down by shRNA (sh) in 3 independent clones (.1, .2, .3) are also reported. Finally, mean of the ratios (log2r.mean) and p-values of paired t-test in the 8 independent experiments are highlighted in yellow. Only genes with significant p-values are indicated. A list of the genes without significantly different expression is available upon request. Cellular models-pathways worksheet bar: pathways with enrichment in cells with or without IGF2knock-down. Up genes (in red): genes up-regulated when IGF2 is knocked-down. Down genes (in green): genes down-regulated when IGF2 is knocked-down. In the “Summary” column, the enrichment in up or down-regulated genes of the pathway in cells without IGF2 extinction (IGF2-high ACC cells) is specified. A list of the pathways without enrichment in IGF2-high or IGF2-low cells is available upon request.
(XLS)

Table S5 **IGF2 targets in adrenocortical carcinoma and H295R cells.** This table shows the genes regulated in the same way (up or down-regulated) in tumors and cellular models. log2(ratio IGF2-/IGF2+): mean of log2 ratios of the 8 experiments in cellular models (see table S3). PairedTTest.pvalue: p-value of the paired t-test performed on the 8 independent experiments in cellular models (see table S3). moderate T-test p.values: p-values of the Limma test performed between IGF2_high and IGF2-low carcinoma. log2(Fold change IGF2-/IGF2+): log 2 of fold change, IGF2-low carcinoma over IGF2-high carcinoma. In red: genes positively correlated to IGF2. In green: genes negatively correlated to IGF2.
(XLS)

Table S6 **Quantitative expression of IGF2 signalling pathway, cell cycle and growth factor genes in IGF2-low and IGF2-high adrenal carcinoma.** Results for different genes of the IGF2 pathway are presented, as determined by qRT-PCR (mRNA level) or western blot (protein level). Medians for IGF2-high and IGF2-low carcinoma, as fold change and p-value between the 2 groups are indicated. In bold: significant results.
(XLS)
