# Supplementary figures and images for: IGF2 Promotes Growth of Adrenocortical Carcinoma Cells, but Its Overexpression Does Not Modify Phenotypic and Molecular Features of Adrenocortical Carcinoma
Source: PLoS One. 2014 Aug 4;9(8):e103744. doi: 10.1371/journal.pone.0103744 (PMC4121173; doi:10.1371/journal.pone.0103744)

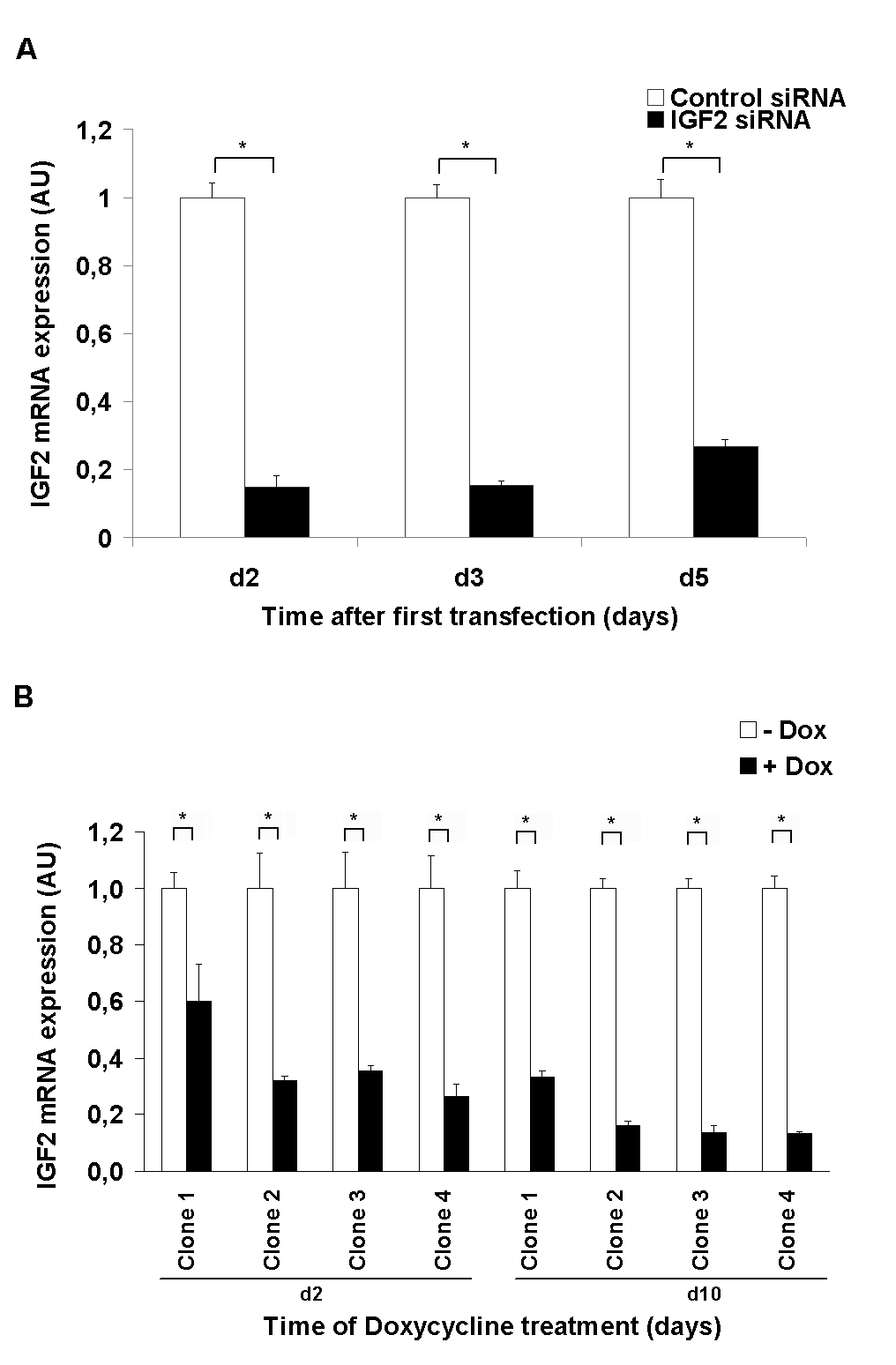

Supplement: Figure S1 — Cellular models of IGF2 knock-down in adrenal carcinoma. A: Transient extinction of IGF2 expression by siRNA in H295R cells. Cells were transfected twice at day 1 and 2 after plating, either with siRNA against IGF2 (black bars) or with control siRNA (white bars). Levels of IGF2 mRNA were measured by qRT-PCR at days 2, 3, and 5 after the first transfection. IGF2 expression was reduced by 85% at days 2 and 3, and 74% at day 5. B: Long-term extinction of IGF2 by shRNA in H295R cells. Levels of IGF2 mRNA were measured by qRT-PCR after 2 (d2) or 10 (d10) days after doxycycline treatment (black bars), and compared with those obtained in the absence of doxycycline (white bars). Results are presented here for 4 different clones, with 40 to 75% (d2) or 65 to 90% (d10) reduction of IGF2 expression. PP1A gene is used as a reference in both experiments and expression ratio between IGF2 and cyclophilin has been considered as 1 for the control experiment (control siRNA for A and no doxycyclin for B). Results were analysed using Wilcoxon test. *: p-value<0.05 Results are representative of at least 3 independent experiments. (TIF) [file pone.0103744.s001.tif]

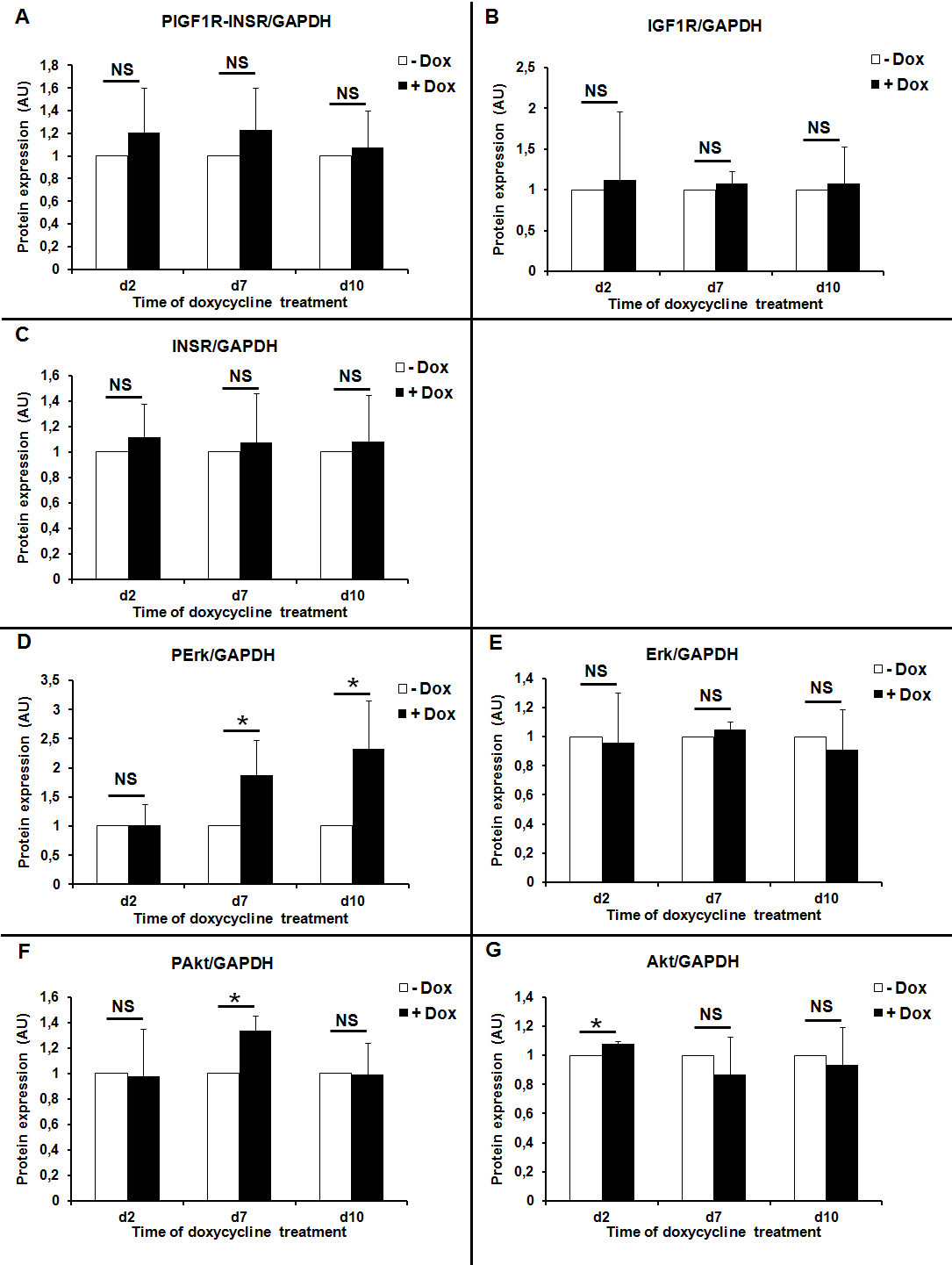

Supplement: Figure S2 — Study of IGF2 receptors, Erk, and Akt activation by western blot after 2, 7, or 10 days of IGF2 knock-down in stably transfected clones. For each clone, expression of proteins was normalized to GAPDH expression, and fixed to 1 in the absence of doxycycline (white bars). Means and standard deviations of these ratios for 3 different clones after doxycycline treatment are indicated (black bars). A, D, F: Activated proteins as determined by their level of phosphorylation. B, C, E,G: Total proteins A, B, C: IGF2 receptors. D,E: Erk. F,G: Akt. Results were analysed using Wilcoxon test. *: p-value<0.05. (TIF) [file pone.0103744.s002.tif]
